# Collectanea

**Published:** 1830-03

**Authors:** 


### COLLECTANEA.

Floriferis ut apes in saltibus omnia libant,
Omnia nos, itidem, depascimur aurea dicta.

### PATHOLOGY.

*Fatal Hemorrhage from Rupture of Varicose Veins in the Legs.*

VARICES of the legs are usually considered rather as a source of inconvenience than of positive danger. The two following cases, although similar instances are certainly uncommon, may tend to diminish the confidence both of the surgeon and the patient under such circumstances. They also show, better than any abstract reasoning, the necessity of habitual compression of the extremities affected with a varicose state of the veins.

*Case by* M. REIS. A pavior was attacked, whilst at work, with hemorrhage from the left leg, and in ten minutes he died, notwithstanding compression was immediately applied. Upon examination after death, a varicose ulcer, from ten to twelve lines in diameter, was discovered in the leg, in the middle of which one of the superficial veins had ruptured.

*Case by* M. FORESTIER. A woman, in the seventh month of pregnancy, was suddenly attacked with violent hemorrhage from the leg. M. F. was sent for, and found the blood spouting forth with extraordinary violence from a varicose vein which had burst. A finger was instantly applied to the wound, and the bleeding was arrested by compression. The quantity of blood lost by this patient was enormous: the chamber was perfectly inundated by it. For many days she remained very weak: she gradually, however, recovered her strength, and her labour took place at the usual period, and terminated favorably. If in this case immediate assistance had not been afforded, death must speedily have ensued from the excessive violence of the bleeding.—*Journ. gen.*

---

*Disorganization of the Cuticle in New-born Infants.*—A very important disease of the skin of the fœtus consists in a peculiar condition, causing the epidermis to peel off the soles of the feet, the toes, the palms of the hands, the fingers, and sometimes the whole body, on the slightest touch. I have only once seen the cuticle peeling off in this manner from the whole body and limbs, in a perfect living child ; but I have more frequently seen it take place in the palms of the hands and the soles of the feet. In every case it was tolerably clear that the mother had suffered from syphilis during her pregnancy. None of the children lived more than a few days, and, though not born before their time, they were meagre and weakly. The spots from which the cuticle had separated became inflamed, which contributed, together with the general debility of the infants, to the speedy termination of their existence. I am still in the dark as to the nature of this disease.

This condition of the skin on the hands and feet was several times accompanied by an eruption on the same parts, of a variolous character, yet differing from the smallpox in the form and appearance of the pustules. It bore the nearest resemblance to cowpox on the twelfth or thirteenth day of the eruption. I do not know any more of the nature and course of this exanthematous disease, because all the children afflicted with it died soon after their birth, and thus rendered further observation impossible.—Jörg *über die Kinderkrankheiten*, §§ 310, 311.

---

*Remarkable Abscess communicating with the Caput Coli.*—A young man, aged nineteen, on the evening of 16th September, 1827, was seized, after eating freely of pears, with pain of the bowels, accompanied with much vomiting and purging. These symptoms were relieved by the usual means, but were immediately followed by fixed pain in the right iliac region, a little below and inwards of the superior spinous process of the ilium. At first nothing unusual was discovered by examination of the part; but after a few days, a deep-seated circumscribed swelling, about the size of an egg, was felt: it was exceedingly painful to the touch, and gave much pain in motion, but the skin covering it was healthy. The functions of the stomach and bowels were now in a natural state, but there was much fever with high delirium. General and topical bleeding, and all the other usual remedies, which were now carefully administered by Dr. Begbie, failed in giving any relief. Fever continued with high delirium ; the swelling was still very tender to the touch, and there were frequent attacks of strong rigors. In the beginning of October, the swelling became more diffused and less painful, and an obscure feeling of fluctuation was discovered in it. On the 3d, he was seized with severe diarrhœa, accompanied by a tympanitic state of the abdomen; the local affection then became less urgent, but the constitutional symptoms continued, and assumed the characters of the advanced stage of low fever, and he died, gradually exhausted, on the 14th.

*Inspection.* Immediately above the caput coli, the omentum had contracted a very firm adhesion to the ascending colon and to the parietes of the abdomen; and in this manner was formed a circumscribed cavity, bounded by this portion of omentum, the posterior surface of the caput coli and the portion of peritoneum lining the parietes at the part. This cavity contained a small quantity of ill-conditioned pus, and three or four bodies, which were found to be the seeds of fruit, covered by an earthy incrustation; it communicated with the caput coli by a small irregular opening, and the mucous membrane around the opening was thickened and highly vascular. The cavity of the abscess was also found to extend behind the peritoneum covering the iliac muscles, and upwards, along the whole extent of the lumbar vertebræ.

There is an obscurity in the pathology of this singular case; and it seems difficult to say whether the abscess had been originally formed and had burst into the caput coli, or whether the perforating ulcer of the caput coli had been the primary disease, and the escape of its contents had given rise to the abscess. The existence of the seeds of fruit, covered by an earthy incrustation, in the cavity of the abscess, would appear to favor the latter supposition. —ABERCROMBIE *on Diseases of the Stomach.*

---

*Extensive Disease of the Rectum and Prostate Gland: Stricture of the Arch of the Colon, &c.* A gentleman, aged seventy-two, had been liable for fifteen years, to frequent desire to pass urine, which generally obliged him to get up five or six times in a night, and it was usually accompanied at each time by a desire to go to stool. This at last increased to such a degree, that, for several years before his death, he scarcely ever made water without having his bowels moved. His general health, however, continued good, until about a year before his death, when he began to fall off greatly in flesh and strength. Soon after, his legs became œdematous and his pulse feeble, and he was greatly distressed with flatulence. The frequent desire to pass urine continued, but it was passed without pain. On examination, the prostate was found so much enlarged as to prevent the passage of the finger into the rectum. The abdomen was now tense and tympanitic, and hard deep-seated ulcers were felt in various parts of it, especially in the left side, where they were painful on pressure. The bowels continued quite open or easily regulated, and his motions were of a healthy appearance and rather fluid. He died, gradually exhausted, in July 1827.

*Inspection.* The prostate was very much enlarged, and of a soft cheesy consistence, so that it broke down under slight compression. The coats of the rectum were much thickened, and it adhered extensively to the neighbouring parts. The sigmoid flexure of the colon adhered to the brim of the pelvis. The bladder was much thickened and contracted, but its internal surface was healthy. In the caput coli there was a small ulcer, and in the right side of the arch of the colon there was a thickened and contracted portion, about an inch in extent, which admitted only a small finger. The other parts of the colon, both above and below this contraction, was distended with large hard masses of feculent matter, many of them the size of large eggs; and it appeared that they had formed the tumors which were felt during the life of the patient.

It is unnecessary to point out the pathological facts which are illustrated by this case. One not unworthy of attention consists in the masses of hard feces in the colon, assuming, in a great degree, the characters of glandular tumors, and some of them being even painful on pressure. It also illustrates, in a striking manner, that singular state of the bowels, in which fluid feces may be discharged regularly and freely, and apparently in abundant quantity, while there is going on for a length of time an immense accumulation of feculent matter, in a very hardened state, extending through the whole of the colon. —*Ibid.*

---

*Pathological Researches on Inflammation of the Veins of the Uterus, with additional Observations on Phlegmasia Dolens.* By ROBERT LEE, M.D. Physician Accoucheur to the British Lying-in Hospital. (*Medico-Chir. Transactions,* vol. xv. part ii.)

In a former communication, which I had the honour of presenting to this Society, on the Pathology of Phlegmasia Dolens, I was led, from a series of facts, to infer that inflammation of the iliac and femoral veins gives rise to all the phenomena of that disease in puerperal women. Subsequent dissections have enabled me not only to confirm the accuracy of my former observations, but have led me to discover the important pathological fact that, in phlegmasia dolens, the inflammation commences in the uterine branches of the hypogastric veins, and subsequently extends from them into the iliac and femoral trunks of the affected side.

The object of the present communication is to submit to the consideration of the Society the various facts which appear to establish the truth of these general views of the nature and origin of phlegmasia dolens; and to detail the observations I have made on inflammation of the uterine veins.

CASE I. *Phlegmasia Dolens, followed by the usual symptoms of phlebitis and death. The principal abdominal veins, and those of the right inferior extremity, inflamed and obstructed.*

Mrs. Edwards, æt. thirty-five; No. 54, King street. 16th of April, 1829, was delivered of her second child, three weeks ago, after a natural labour; and on the 9th instant was attacked suddenly with pain in the calf of the right leg, and loss of power in the whole right inferior extremity.

On the 13th, a considerable swelling, without discoloration, had taken place from the ham to the foot, and great tenderness was experienced along the inner surface of the thigh to the groin.

The extremity is now universally swollen, painful, and deprived of all power of motion. The temperature along the inner surface of the limb is increased; the integuments are pale and glistening, and do not pit upon pressure. There is no pain in the hypogastrium, but pressure along the course of the crural vessels excites great suffering, and the vein from the groin to the middle of the thigh is indurated, enlarged, and exquisitely sensible. There is also great sensibility in the ham, and along the inner surface of the leg to the ankle, where some branches of the superficial veins are hard and painful on pressure. Pulse eighty; tongue much loaded; thirst; bowels open. There was no rigor or symptom of pyrexia at the invasion of the disease. She states that the veins of the right extremity were more distended during pregnancy than those of the left.

Twelve years ago, after the birth of her first child, the patient and her relatives report that she experienced an attack similar to the present, in the same limb; and that it remained in a weak condition for several months afterwards, but ultimately recovered its natural size and power.

18th April.—The tension and increased heat along the inner surface of the limb are somewhat diminished, but the pain continues in the course of the vessels.

May 1st.—Affection declining. The femoral vein cannot now be felt, but there is still a sense of tenderness in its course down the thigh. No pitting on pressure. She has suffered, during three or four days, considerable uneasiness between the umbilicus and pubis, as well as in the loins, and has had rigors, with quick pulse, loaded tongue, and thirst. The abdomen is soft, but tender on pressure around the umbilicus.

9th.—The swelling of the limb is nearly gone, as is the tenderness in the course of the femoral vessels. For several days past, she has experienced attacks of acute pain in the umbilical region, loins, and back, which have assumed a regular intermittent form. Every afternoon there has been a violent rigor, of an hour's duration, followed by increased heat and profuse perspiration. In the course of the last and preceding nights, there was slight delirium. The skin is now hot and dry, the pulse 125, the tongue brown and parched, bowels open.

The abdomen is neither tense nor swollen. On pressing around the umbilicus, she complains of a deep-seated feeling of soreness. A strong vibratory motion, corresponding with the pulsations of the heart, is perceived in the epigastrium.

21st.—The febrile attacks gradually declined in severity, and she appeared to recover until yesterday, when she had a long and violent fit of cold shivering. The countenance is now expressive of great anxiety, and the pulse extremely quick and feeble. There remains no visible trace of the affection of the lower extremity.

23d.—Has been vomiting ever since yesterday, at intervals of half an hour. Complains of great pain in the left side, increased upon taking a deep inspiration. The pulsation in the epigastrium diminished, although it is still clearly perceived; pulse 120, and soft.

24th.—Symptoms continue without alleviation. Has had a severe shivering fit of long duration. Skin hot and dry; pulse 140; tongue brown and parched; diarrhœa. The pulsation in the epigastrium has entirely disappeared; the pain in the left side of the thorax is diminished; but the respiration is hurried, and there is frequent cough. Great prostration of strength. Surface of the body has assumed a peculiar sallow tinge. She has been delirious in the night, but is now perfectly conscious when roused.

The conjunctiva of the right eye has suddenly become of a deep red colour, and so much swollen that the eyelids cannot be closed. The cornea is dull; she makes little complaint of pain of the eye, and there is no intolerance of light. The vomiting has ceased.

25th.—Has again had repeated attacks of vomiting. Debility rapidly increasing; respiration hurried; incessant hacking cough; pulse 140, extremely feeble; surface of the body cold and clammy; the tongue and teeth covered with dark sordes; diarrhœa. The left eye has also become red and swollen, without much increased sensibility.

26th.—Great debility. When undisturbed she is delirious, but is conscious when roused, and complains of pain in the left side of the chest. Pulse above 140; tongue black and dry. Conjunctiva of left eye also affected with swelling and intense redness. The cornea is dull, and shreds of lymph appear to have been effused over the left iris.

28th.—Had so violent a rigor in the afternoon that the bed shook under her. She is now completely insensible. The eyes are red and swollen, and there is a copious secretion of an opaque fluid from their surface and from the eyelids, which cannot be closed. The respiration hurried; pulse 140.

31st.—Has recovered her consciousness, and drank cider and porter with great eagerness. Pulse rapid and feeble. Eyes so much swollen that they seem pushed forward from their orbits. Vision entirely lost; but hearing and the other senses remain.

2d June.—Great debility. A red puffy swelling has suddenly appeared over the right elbow-joint. Tongue dry and black; diarrhœa; frequent, or rather constant wandering, except when spoken to, when she answers questions distinctly, and complains only of pain in the chest, with difficult respiration and cough.

10th.—Little change has taken place in the symptoms, but she has become much weaker. The vision is lost, but the hearing is perfect, and she makes no complaint of pain in any part of the body.

15th June.—Died this morning.

*Morbid appearances on examining the body of Sarah Edwards, on the 16th June.* Present, Drs. Sims and Locock.

Thorax: In its left cavity were contained upwards of two pints of a thin purulent fluid, and extensive recent adhesions existed between the pleura covering the lower margin of the superior lobe, and the pleura costalis. The surface of the inferior lobe was coated with a thick layer of flocculent coagulated lymph, as was a corresponding part of the pleura costalis. The substance of this lobe was of a dark colour approaching to black, and soft in texture, so as to be readily broken down with the fingers. In its centre about an ounce of thick cream-coloured pus was found deposited in the dark-coloured and softened lung. This was not contained in any cyst or membrane, but infiltrated into the pulmonary tissue.

In the right cavity of the chest, recent adhesions also existed at the inferior part. A considerable portion of the right inferior lobe was entirely changed from the healthy structure, being converted into a dense, solid, dark red-coloured mass. On the anterior surface of this lobe, the pleura was elevated as if by a hard irregular tumor, but, when cut into, no pus escaped from this part, and it presented only the appearance of the surrounding portions of lung with a greater degree of condensation.

Vena cava inferior: Coats of the vessel considerably thickened, and the internal, where visible, of a scarlet colour; its whole cavity occupied by a coagulum, distending it to its utmost extent, and terminating in a loose pointed extremity, about an inch below the entrance of the vena cava hepatica. The coagulum, covered with a membranous-like investiture of a bright red colour, throughout firmly, and in many places inseparably, adherent to the inner lining of the vein; the substance within it varied in consistence and colour; in some parts it presented the appearance of coagulable lymph, in others it was a pultaceous dull yellow mass, made up apparently of pus and lymph blended together. The exterior of the firmer portions were separated into layers, which gradually diappeared as they approached the centre. The mouths of all the veins emptying themselves into the cava were sealed up, the emulgents excepted; the coagulum, near the entrance of these vessels, hanging loosely within the cava.

Left common iliac and its branches: Its interior plugged up with a continuation of the coagulum from the cava, and differing in no respect from it, either as to consistence, colour, or the firmness of its adhesions to the inner tunic of the vein; it was continued beyond the entrance of the internal iliac, (which it completely closed,) and terminated in a pointed extremity about the middle of the external iliac. Neither the remainder of this vessel nor the femoral vein exhibited any morbid changes. The internal iliac was much contracted, and lined with a thick adventitious membrane.

Right common iliac and its branches: This vessel was contracted to more than one half its natural size; it was firm to the touch, and of a grayish blue colour; to its internal coat adhered an adventitious membrane of the same colour, containing within it a firm coagulum, made up of thin layers of dense lymph. The internal iliac was rendered quite impervious by dense, dark-coloured, bluish membranes, and at its entrance into the common iliac was converted into a solid cord.

The contracted external iliac contained within it a soft yellowish coagulum, similar to the one in the cava; its coats were three or four times their natural thickness, and lined with dark-coloured membranous layers.

The femoral vein, from Poupart's ligament to the middle of the thigh, was diminished in size, and almost inseparable from the artery. Its tunics were thickened, and its interior coated with a dense membrane surrounding a solid purple coagulum strongly adherent to it. The superficial and deep femoral veins were in a similar condition, and the saphena major and minor differed from the femoral veins only in the size of the coagulum they contained, which was slender, and had formed no adhesions with the layers of lymph lining their cavity.

The cellular membrane and other textures of the limb were in a perfectly healthy condition, and in size and appearance there was externally no visible difference between the two extremities.

The morbid alterations of structure now described can still be distinctly seen in the preparation of the diseased veins, and have been represented with great accuracy in the beautiful drawing made by Mr. Perry from the parts, immediately after their removal from the body.

CASE II. *History of a Patient who died of Tubercular Phthisis, subsequent to an attack of Phlegmasia Dolens.*

Mrs. Foster, æt. fifteen, No. 27, Little Windmill street, out patient of the British Lying-in Hospital.

May 8, 1829.—Previous to her confinement, six weeks ago, she had been affected for several months with pain in the chest, difficulty of respiration, cough, with copious expectoration of a matter tinged with blood, profuse perspirations in the night, and had become greatly emaciated.

During the last fourteen days, she has been suffering from attacks of pain in the bowels and diarrhœa.

On the 4th instant, she experienced a sense of soreness in the left groin, which gradually extended along the inner surface of the thigh to the ham, and from thence along the posterior surface of the leg to the foot. She stated that, for two days before the occurrence of pain in the groin, she had felt great uneasiness in the region of the uterus; that this suddenly quitted the hypogastrium, and passed into the groin; and that from thence it extended downward along the inner surface of the thigh and leg. The limb became swollen twenty-four hours after the invasion of the pain.

The whole left inferior extremity is now affected with a hot, painful, colourless swelling, no where pitting on pressure, except over the foot. The thigh is double the size of the other, and any attempt to move the limb produces excruciating pain along the inner surface of the thigh; and the pain excited by pressure along the tract of the femoral vein is so acute that the condition of this vessel cannot be ascertained. Several branches of the saphena major above the knee are distended and hard; pulse 120; respiration quick and laborious; tongue peculiarly red and glossy; diarrhœa continues.

10th.—Pulmonary affection aggravated. The limb continues extremely painful, and is still more swollen. The groin is so tender that she cannot endure the slightest pressure over it. The same is the case with the inner surface of the thigh. The branches of the saphena vein are still hard and painful.

11th.—The femoral vein, under Poupart's ligament, can now be felt indurated and enlarged, and it is exquisitely painful when pressed; as is the inner surface of the thigh, the ham, and the calf of the leg. There is comparatively little tenderness along the outer surface of the limb; pulse 120; skin hot.

17th.—Diarrhœa, emaciation, colliquative sweats, and difficulty of respiration increasing. The left inferior extremity is still much swollen, but there is less pain at the groin, and in the course of the femoral vessels. The foot and ankle pit on pressure.

26th.—Calf of the leg still swollen and painful.

June 19.—The pulmonary affection aggravated, and she is now reduced to a state of extreme debility. The limb is still considerably swollen, and is universally œdematous.

24th.—Died this morning.

*Dissection.* Present, Dr. Sims and Mr. Prout.

Thorax: Extensive adhesions between the pleuræ on both sides. Scarcely a portion of lung could be observed which did not contain tubercles, in various stages of their growth. The right and left superior lobes contained several large tuberculous excavations.

The vena cava and right common and external iliac veins were in a sound state.

The left common external and internal iliac veins were all impervious, and had undergone various alterations of structure.

The common iliac, at its termination, was reduced to a slender tube, about a line in diameter, which was lined with a bluish slate-coloured adventitious membrane. The remainder of the common and the external iliac veins were coated also with a dark-coloured membrane, and their centre filled with a brownish ochrey-coloured tenacious substance, rather more consistent than the crassamentum of the blood.

The left hypogastric or internal iliac vein was in the same condition, but in some places reduced to a cord-like substance, and its cavity throughout completely obliterated. The branches of this vein taking their origin in the uterus, and usually termed the uterine plexus, were found completely plugged up with firm reddish coagula of lymph. From the commencement of the branches of this plexus of the hypogastric vein to the termination of this vein in the iliac, the whole had become thickened, contracted, and plugged up with coagula and adventitious membranes of a dark blue colour.

The same changes had taken place in the uterine plexus and trunk of the right hypogastric vein, from the uterus to its unusual termination in the left common iliac vein.

The coats of the left femoral vein were thickened, and closely adherent to the artery and surrounding cellular substance; its whole interior lined with an adventitious membrane, and distended with a reddish-coloured coagulum. The same morbid changes presented themselves in the deep and superficial branches, as far as they were examined down the thigh.

*** Dr. Lee's paper, from which we have taken these cases, must be considered one of the most valuable pathological contributions of the day. In our opinion, Dr. L. has clearly shown the true pathology of phlegmasia dolens. We confess we were before doubtful of the connexion of this disease with inflammation of the veins; but Dr. Lee has entirely removed our scepticism upon this point.—Editor.

---

### PRACTICAL MEDICINE.

*Arsenic in a Case of Leprous Affection and Periodical Headach.* In the third volume of the Philadelphia Medical Museum, a highly interesting case of leprous affection, combined with periodical headach, is related by Dr. J. R. Coxe, in which the value of arsenic was very conspicuous, and in which any excess over fifty drops of Fowler's solution was followed by unpleasant consequences. The case also illustrates the safety of the article, as the patient commenced with twenty drops three times a day, gradually increasing the dose, till, at the end of six weeks, she took fifty drops three times a day, and that for several weeks! For a short time she took sixty drops three times a day, but this disagreed, and she returned to fifty. She took altogether, in the course of somewhat less than four years, between a quart and three pints! The remedy had a beneficial effect on both complaints, which returned, however, from time to time when the medicine was laid aside, but always with diminished intensity.

---

*Utility of Ipecacuanha in Hæmatemesis.** (Dr. Condie *on Hæmatemesis. North American Med. and Surg. Journal.)*

In the summer of 1827, we were requested to see a young woman who was vomiting blood. The patient was about eighteen years of age; she had suffered for a long time from attacks of intermittent fever, which had been finally suspended by large doses of the bark. She did not, however, recover her health, but remained in a state of langour, without appetite; her bowels

* To this practice the attention of the profession was first directed by Dr. Sheridan. In a paper he published in the fourth volume of the Transactions of the Dublin College of Physicians, he reports five cases of hæmatemesis, in which Ipecacuanha was employed with the best effects.

constipated; the skin of a yellow hue, dry and harsh; her tongue foul. Occasionally she felt chilly, and was immediately afterwards flushed with heat. She complained of pain in the head, and disinclination to motion. Menstruation was suspended; some tumefaction was experienced in the right hypochondrium, with tenderness of the epigastrium, and frequently violent attacks of gastralgia. When we saw her, she was discharging vast quantities of dark-coloured blood, partly fluid and partly coagulated; her face was pale, pulse small and feeble, tongue pointed and red at the edges. Twenty grains of ipecacuanha were directed in one dose. This was taken late in the afternoon, and produced considerable vomiting, first of pure blood, then bloody mucus, and finally mucus alone. She now fell asleep, and awoke the next morning without nausea, but much exhausted. Her bowels were directed to be opened by an enema, and toastwater alone for food and drink. There was no return of the hemorrhage. In a few days, remedies adapted to the morbid condition of the stomach and liver were entered upon; and the patient finally recovered, and has remained perfectly well to the present day.

In the spring of the same year, we had under our care, with vomiting of blood, a female, forty-six years of age, of a robust habit and dark complexion. She had suffered for some time previous from irregularities of the menstrual flux, with a feeling of weight and uneasiness at the præcordia; occasionally dull pains in the epigastrium; frequent chills, followed by a burning heat of the skin; lowness of spirits, and deficient appetite. For the few days before our visit, she had frequently thrown up a quantity of blood by vomiting, and was then discharging it profusely. The pulse was contracted; skin dry and harsh, its temperature being somewhat increased; tongue furred in the centre, very red at the margin. Directed a scruple of ipecacuanha to be divided into four powders, one of which to be administered every hour. After the first had been taken, the vomiting was suspended: the remedy produced neither nausea nor vomiting. The ensuing day the patient was confined to toastwater; twelve ounces of blood were taken from the arm, and a purgative of calomel administered. The hemorrhage did not return, and an appropriate treatment very soon removed all symptoms of gastric disease. The patient has since enjoyed uninterrupted health.

A female, forty-three years of age, during the summer and autumn of 1828, had suffered from frequent attacks of fever and ague. Emetics, purgatives, arsenic, and quinine, were the remedies employed for its cure. The regular paroxysms of the fever had by these means been suspended; but the patient was left in a state of great muscular weakness, disinclined to motion, without appetite, and depressed in spirits. Her bowels were constipated; she was subject to frequent rigors, succeeded by flushes of heat, and a burning in the palms of the hands; and there was considerable tumefaction of the abdomen, tenderness, with dull constant pain of the epigastrium, and great thirst. About the 10th of January last, she was attacked with hæmatemesis: the discharge of blood was profuse, and when I saw her she presented all the appearances consequent upon excessive hemorrhage. A scruple of ipecacuanha in four powders, one to be taken every hour, was directed. Soon after the powders were commenced with, the hemorrhage ceased; but great thirst and nausea still continued. The next day the vomiting of blood returned, but not to so great an amount. Twenty grains of ipecacuanha were given at once, and a considerable quantity of mucus coloured with blood was brought up, when the hemorrhage and vomiting ceased. About two weeks subsequently, the discharge of blood returned, but was immediately suspended by small doses of the ipecacuanha; three grains every hour. Notwithstanding every remedy subsequently employed in this case, the disease of the stomach and liver was of such a nature that the recovery of the patient has been extremely slow. As the gastric symptoms by degrees abated, an ascites was gradually developed, from which she is now rapidly recovering; and I have every prospect of being shortly able to restore her to perfect health.

Equally efficacious with the ipecacuanha, in the suspension of stomachic hemorrhage, we have found to be the administration of minute doses of calomel; say from one quarter to half a grain every hour. The remedy in these doses we have repeatedly employed, and never without the desired effect. Whether there is any difference in the set of cases to which one or other of the foregoing plans of treatment is particularly adapted, we have ourselves not been able to determine. The small doses of calomel will, of course, be preferred, whenever it may be deemed improper or inexpedient to risk the production of vomiting; an effect which will frequently result from even inconsiderable doses of the ipecacuanha: but in all cases in which the hemorrhage is profuse, and it is apprehended that the patient may speedily sink under it, we would prefer the latter, in consequence of the greater promptness with which it acts in suspending the effusion of blood.

After the violence of the hemorrhage is abated, we have experienced beneficial effects, in cases connected with hepatic disease, from the application of a blister in the neighbourhood of the liver.

In hæmatemesis occurring in females between eighteen and thirty years of age, preceded by great languor and oppression about the chest and præcordia, a sense of fulness in the latter, cough, dyspepsia, and sometimes pain in the breast, loss of appetite, headach, vertigo and disturbed sleep, a dulness of the eyes, and a countenance expressive of great distress, a feeble pulse, and constipated bowels, Dr. HAMILTON has found, he declares, that the proper exhibition of purgatives, so as to produce a free evacuation from the bowels, affords an effectual means of removing the hemorrhage. "Suspended menstruation," remarks Dr. H. "is not a necessary concomitant of this species of hemorrhage." Calomel was the purgative generally employed by this gentleman in these cases.

We have also treated stomachic hemorrhage by the administration of scruple doses of calomel; and in some instances with the most prompt and decided good effects. In general, however, we prefer the employment of the calomel in the small doses already indicated. From any other of the list of purgatives we should anticipate harm rather than advantage in hæmatemesis.

---

*Specific for the Hooping-cough.* In RUST's *Mag. f. die gesammt. Heilkunde*, (No. ii. 1828,) it is stated that Dr. MEYER, of Minden, has in a few days been enabled to remove all the symptoms of pertussis, by the external application of morphia. He directs a small blister to be applied over the præcordia; the detached cuticle being removed, the exposed surface is to be sprinkled over with half a grain of morphia, rubbed up with starch. The morphia is to be repeated every evening. The only internal remedy he employed was an emetic. If necessary, the blister may be reapplied every third day. In five cases, the disease was so far diminished in eight days, that no further treatment was considered necessary.

*Chlorine in Hydrophobia.* M. JULIA FONTENELLE, in one of his clinical lectures, after mentioning the experience of Drs. SCHOENBERG and SEMMOLA in favor of chlorine applied to the wound, and taken by the mouth, diluted in water, as a cure for hydrophobia, thinks that the chloride of soda might be advantageously substituted internally for the chlorine. M. COSTER strongly recommends the application of chloride of lime to the wound, as capable of completely neutralizing the poison.

---

### SURGERY.

*Solution of Nitrate of Silver in Diseases of the Eye.* The *Journal für Chirurg. und Angenheilkunde*, vol. xii. (No. i. 1828,) contains an abstract of Professor GRAEFE's clinical report of the Institute for Surgery and Diseases of the Eye of the University of Berlin, for the year 1827. In this, among other interesting particulars, it is mentioned that the efficacy of a solution of the nitrate of silver, dropped into the eye, or applied by means of a hair pencil, as a remedy in obstinate cases of Blennorrhagia Oculi, the employment of which was noticed in a preceding report, has been confirmed by the experience of the present year.

---

*Gonorrhœa cured by Injections of Balsam Copaiba.* Dr. ELIJAH COONS, of Tuscumbia, Alabama, records, in the Transylvania Journal, the account of a case of Gonorrhœa, (which ought rather to be called gleet or chronic gonorrhœa,) cured, after other remedies had failed, by the use of balsam copaiba used as an injection four or five times a day. Diet spare; drinks mucilaginous; Seidlitz powders used to keep his bowels open. In a period of between two and three weeks he was quite well. Dr. C. had introduced a bougie into the urethra, but did not detect any stricture.

He has since had two cases similar to the first, but of shorter duration, in which the same happy effect followed the above treatment.

---

### MIDWIFERY.

*Dropsy of the Allantoid.* I have few anomalies to mention relating to the allantoid, that pouch between the amnion and the chorion; and it is probable that this organ is subject to few irregularities, because, in the human species, it is in a state of activity only during the first three or four months of pregnancy, and afterwards sinks into repose. Nevertheless, I have observed that, during the latter months of gestation, a large quantity of fluid is sometimes collected in it, without injury, however, to the fœtus or the mother, as the fluid is discharged every fortnight or month; thus relieving both the woman and the child. Were it not for this, as the dropsy of the allantoid is sometimes very considerable, the woman's health would be endangered by an over-enlargement of the uterus.—JÖRG *über die Kinderkrankheiten*, § 305.

*** The evacuation of this fluid sometimes gives rise to the alarming suspicion that the membranes have been prematurely ruptured, and that abortion is about to take place; as in the case of Mrs. Finlayson, related by Dr. MERRIMAN, in his Synopsis of Difficult Parturition, fourth edition, p. 223.—EDITOR.

### MATERIA MEDICA.

*Sedative Action of Asparagus upon the Circulation.* Digitalis and prussic acid possess the property of weakening the action of the heart, but the employment of these medicines is frequently prevented by the gastric irritation they produce. M. Broussais proposes, as a substitute for these remedies, asparagus, which is perfectly inoffensive to the stomach, and acts as a sedative upon the heart. If a patient, suffering from hypertrophy and excessive action of the heart, eat asparagus, M. Broussais assures us he will find relief; and if the remedy is discontinued, the habitual symptoms will return. Syrup of the green ends of asparagus, like the plant itself, has the power of diminishing the action of the heart, without annoying the stomach. A man, labouring under hypertrophy of the heart, perceived a very decided alleviation of his sufferings while he was in the habit of eating asparagus, and he consequently prepared a syrup of the plant for use when it was out of season. A physician, whom M. Broussais does not name, but to whom he is indebted for this discovery, collected many cases in support of this statement; and the Professor of Val-de-Grâce declares that it is confirmed by the result of his own experience.—*Ann. de la Méd. Phys.*

*** There is a popular, and perhaps even a professional, opinion that asparagus acts as a diuretic. When eaten in any quantity, this plant certainly imbues the urine with a peculiar odour; but, as far as we have observed, Cullen correctly remarks that "neither the quantity of the urine is increased, nor its quality anywise changed." Boerhaave and Van Swieten thought that, upon some occasions, they had observed the eating of asparagus to hasten fits of the gout. We doubt the correctness of this supposition.—
Editor.

### NATURAL HISTORY.

*Method of killing Insects for Preservation in Cabinets.* This method consists in enclosing the insect in a paper or thin wooden box, and exposing it for one or two seconds to heat near the fire. The heat immediately kills insects the most tenacious of life. The process does not alter the most delicate colours; but if the heat be continued too long, the wings and other parts of the body begin to wrinkle.—*Bull. Univ.*

### MISCELLANEOUS.

*Poisoning by Strychnia.* M. Guibourt stated lately to the Academy of Medicine, that, having observed a dog in violent convulsions, in consequence of having eaten one of the compound balls containing strychnia, or vomica nut, which the police use to destroy wandering animals, he forcibly made it swallow powdered nutgalls, when the muscular convulsions immediately ceased; ipecacuanha was then given to the animal, but the latter could not vomit. The next day, milk was given to it and manna; after which, the dog recovered. M. Caventou said, that the infusion of galls was a very effectual opponent to vomiting, and that he had observed it destroy the power of emetic tartar. M. Orfila has already advised the administration of this infusion in cases of poisoning by opium and salts of morphia.—*Ibid.*

